# Molecules That Have Rarely Been Studied in Lymphatic Endothelial Cells

**DOI:** 10.3390/ijms252212226

**Published:** 2024-11-14

**Authors:** Jürgen Becker, Jörg Wilting

**Affiliations:** Institute of Anatomy and Cell Biology, University Medical Center Goettingen, Georg-August-University Goettingen, Kreuzbergring 36, 37075 Göttingen, Germany

**Keywords:** lymphatic endothelial cell, ANKRD37, CAV1, CAV2, CD59, CNN3, DYSF, KANK3, MARCKSL1, MMRN1, NXN, SPTAN1, SPTBN1

## Abstract

A number of standard molecules are used for the molecular and histological characterization of lymphatic endothelial cells (LECs), including lymphatic vessel endothelial hyaluronan receptor 1 (LYVE1), Podoplanin (D2-40), VEGFR3, Prospero homeobox protein 1 (PROX1), and CD31. The number of molecules whose mutations cause lymphatic malformations or primary congenital lymphedema is considerable, but the majority of these diseases have not yet been characterized at the molecular level. Therefore, there is still considerable scope for molecular and functional studies of the lymphatic vasculature. Using RNASeq, we have previously characterized lymphatic endothelial cells (LECs) under normoxic and hypoxic conditions. We used this information to compare it with immunohistochemical data. We carried out some of the immunohistology ourselves, and systematically studied the Human Protein Atlas, a cell and tissue database based in Sweden. Here we describe molecules that are expressed at RNA and protein levels in LECs, hoping to stimulate future functional studies of these molecules.

## 1. Introduction

The lymphatic vascular system was already known in ancient times, although its functions were misinterpreted. *Venae albae* or *Ductus lactei* were already known to Hippocrates of Kos (460–370 BC), Aristoteles (384–322 BC), and the doctors of the Alexandrian school (approx. 300 BC–600 AC) (cited from [1]). When Gaspare Aselli (re)discovered the lymphatics [2], he knew exactly what he had to look for. He was aware that the *Venae albae* were easy to find in the mesenteries of dogs, for example, but he was probably the first to establish a connection between these milky vessels and food intake. The anatomical representation of the lymphatic vascular system was especially advanced at Italian universities [3], although the function of the system was still very mysterious. One of the first to recognize the importance of directed fluid transport in the lymphatics was Olof Rudbeck [4]. Contrary to the prevailing doctrine, which postulated a lymph flow into the liver, he described the connection to the central venous system. He also recognized a valve system in lymphatics and the coagulability of the lymph.

We should be very humble when evaluating ancient knowledge, because one thing is certain: we are still far from fully understanding the functions of the lymphatic vascular system, although more and more functions are being discussed [5,6,7,8]. The complexity is immediately apparent when we visualize the heterogeneity of lymphatic endothelial cells (LECs) [9] and the long lists of molecules expressed in LECs [10,11], lymph collectors [12], or lymph nodes [13,14]. Development and behavior of LECs is critically regulated by the transcription factor PROX1 [15,16,17], and the histological characterization of human LECs is typically performed with antibodies against PROX1 and CD31 [18], the Vascular endothelial growth factor receptor-3 (VEGFR3, FLT4) [19], the Lymphatic vessel endothelial hyaluronic acid receptor 1 (LYVE1) [20], and the type-I integral membrane glycoprotein Podoplanin (PDPN) [21].

Congenital malformations of the blood vascular system are very common. Those of the lymphatic system are much rarer, mostly located in the head-neck region, and can be life threatening. Most of the lymphatic malformations are caused by somatic mutations in genes involved in the VEGFR3 signaling pathway [22,23,24,25]. However, most genetic causes of lymphatic malformations and primary lymphedema have not yet been discovered. This illustrates that there is still a very large number of molecules whose significance for the development and function of the lymphatic vessels is not yet known. We have previously used RNASeq to study human foreskin-derived LECs under normoxic and hypoxic conditions and defined 162 genes that are significantly regulated by hypoxia [10], as well as highly expressed genes that influence the composition of the extracellular matrix and may be involved in lymphedema-induced fibrosis [26]. Here, we went through the RNASeq list and studied the large number of highly expressed LEC genes. We compared RNA expression with protein expression by systematically studying the Human Protein Atlas [27] (https://www.proteinatlas.org/; accessed on 20 August 2024). In this way, we sought to define additional molecules that can be used for deeper characterization of lymphatic vessels and to define further functions of LECs.

## 2. Results and Discussion

We recently performed expression analyses of three well characterized human foreskin-derived LEC lines under normoxic and hypoxic conditions [10,26] and received a list of approx. 16,000 LEC-expressed genes. Only a very small number of these molecules have been studied in LECs thus far. We have tried to systematically match RNA expression with protein expression using the Human Protein Atlas [27]. According to the Human Protein Atlas, the tissues are normal tissues, though, of course, concomitant diseases of the mostly older donors cannot be ruled out. With the exception of dysferlin, which we studied in combination with the endothelial marker CD31, we relied on morphological criteria to identify lymphatics in tissue sections. We have concentrated on molecules whose function has not yet been investigated in LECs. The number of matches between RNA and protein expression was not very high, but there can be many technical reasons (fixation time of tissue, paraffine permeability of the antibodies, etc.) for this. The expression of the molecules we describe in the manuscript is not restricted to LECs. Some occur in BECs or in other cell types. However, it should be noted that the commonly used LEC markers also occur in various other cell types, and ultimately a selection of molecules will always be necessary to characterize a cell type.

**Figure 1:**
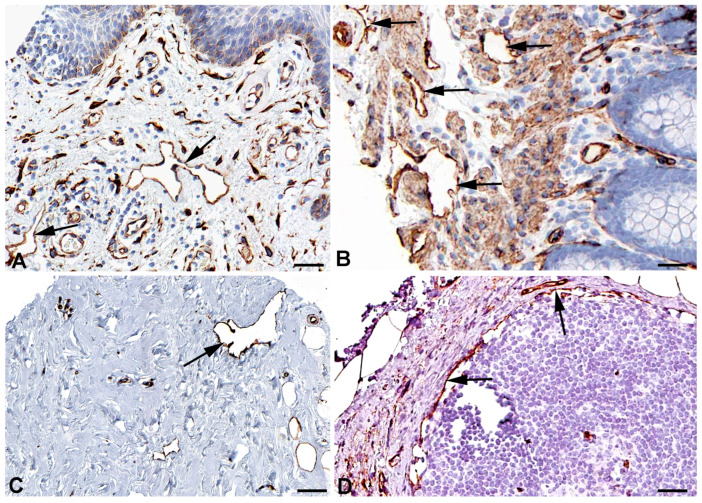
Immunostaining of CAV1 in lymphatics (arrows) of human (**A**) Oral mucosa, Antibody CAB003791 (**B**) Rectum, Antibody CAB003791 (**C**) Breast, Antibody CAB003791, and (**D**) parietal layer of lymph node marginal sinus, Antibody HPA049326. From: The Human Protein Atlas. Bar = 60 µm in (**A**,**D**), and 40 µm in (**B**,**C**).

**Figure 2:**
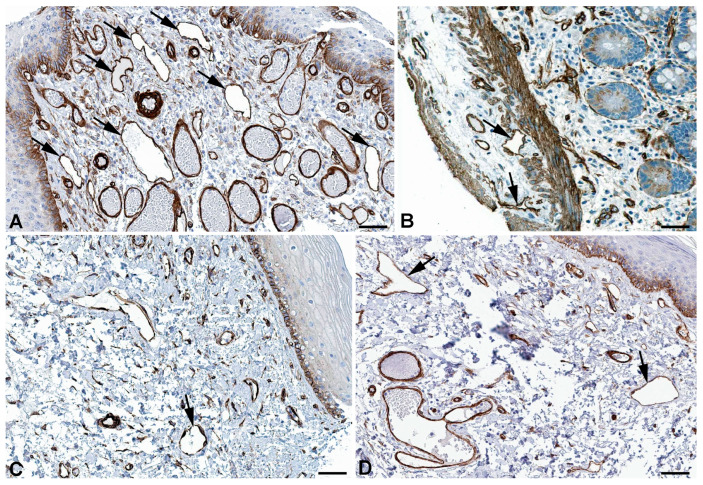
Immunostaining of CAV2 in lymphatics (arrows) of human (**A**) Esophagus, Antibody HPA044810 (**B**) Colon, Antibody CAB013488, (**C**) Oral mucosa, Antibody HPA044810, and (**D**) Skin, Antibody HPA044810. From: The Human Protein Atlas. Bar = 60 µm in (**A**–**D**).

One of the main functions of the lymphatics is regulation of fluid homeostasis. The fluid is mainly absorbed via the flexible microvalves of the initial lymphatic vessels. However, part of the fluid uptake also occurs by means of active transcytosis, which has also been observed for the uptake of chylomicrons by lacteals [28,29,30]. Very recently, the uptake of myofibroblast-derived microvesicles (MVs) was studied in human dermal blood vascular endothelial cells (BECs) and LECs [31]. The authors describe that MVs cross an LEC layer but not a BEC layer in vitro. For both endocytosis and transcytosis, the caveolin-dependent pathway is of utmost importance. High expression of caveolin 1 (*CAV1*) and *CAV2* (Table 1) for endo- and transcytosis seems to be in line with the free passage of MVs through LECs, and antibodies against CAV1 and CAV2 clearly stain lymphatics and selected blood vessels (Figure 1 and Figure 2).

**Figure 3:**
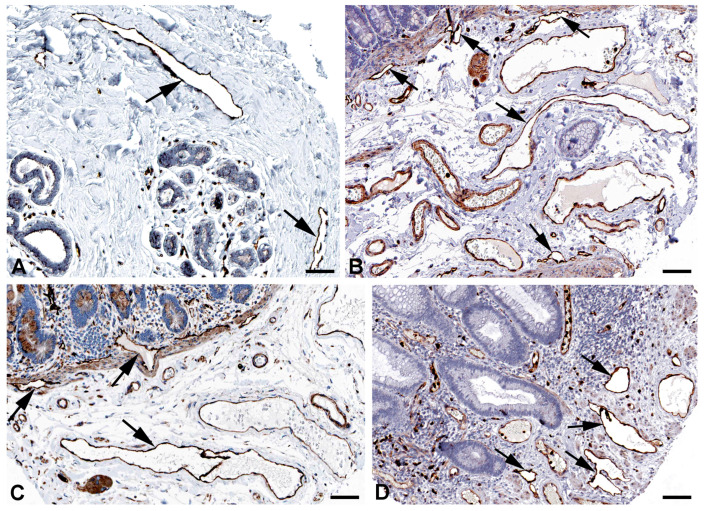
Immunostaining of DYSF in lymphatics (arrows) of human (**A**) Breast, Antibody CAB002510, (**B**) Duodenum, Antibody CAB002510, (**C**) Colon, Antibody HPA017071, (**D**) Skin anal, Antibody CAB002510. From: The Human Protein Atlas. Bar = 70 µm in (**A**,**B**), and 90 µm in (**C**,**D**).

**Figure 4:**
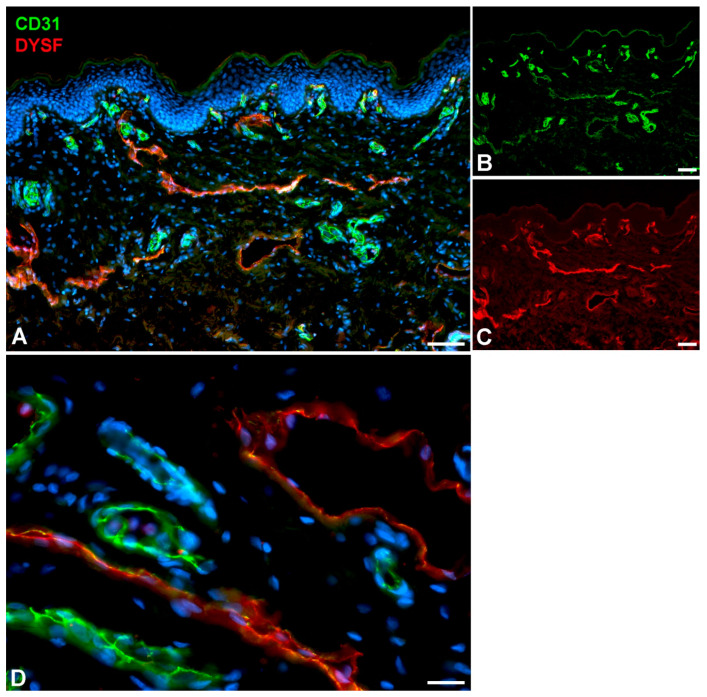
Immunostaining of DYSF (red) and CD31 (green) in lymphatics of human foreskin; overview with epidermis (**A**–**C**) and higher magnification of dermis (**D**). Blood vessels strongly express CD31; lymphatics have a punctate weak staining. Nuclei are stained blue with Dapi. In lymphatics, the red DYSF staining is dominant, but subepithelial capillaries also express DYSF. (**A**–**C**) 10× objective; Bar = 100 µm, (**D**) 40× objective; Bar = 25 µm.

Very active transcytosis by LECs is also reflected by the RNA expression the ferlin family members: myoferlin (*MYOF*; very high), dysferlin (*DYSF*; high), and otoferlin (*OTOF*; moderate) (Table 1). Ferlins regulate membrane fusion and fusion of vesicles to cell membranes e.g., for exocytosis or membrane regeneration [32]. Of the three ferlins, we observed clear immunostaining against DYSF (dystrophy-associated fer-1-like protein) in various organs (Figure 3) and performed immunodouble staining with CD31 in human foreskin (Figure 4). We observed DYSF in dermal lymphatics. Blood vessels were mostly negative, except for the subepithelial capillary plexus. We tested various OTOF antibodies but did not receive a positive result; and, in contrast to the high RNA expression, we found no immunopositivity for MYOF in the Human Protein Atlas. As already mentioned, this apparent discrepancy may be due to technical problems with antibody staining. Antibodies need to be improved and protocols refined. However, DYSF is clearly present in LECs at the RNA and protein levels, which is in line with high transcytotic activity.

**Figure 5:**
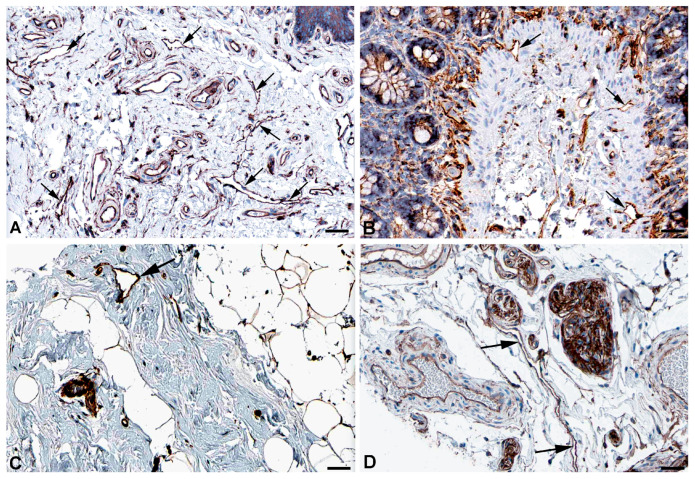
Immunostaining of SPTAN1 in lymphatics (arrows) of human (**A**) Oral mucosa, Antibody HPA007927, (**B**) Small intestine, Antibody CAB004581, (**C**) Breast, Antibody HPA007927, (**D**) Epididymis, Antibody HPA007927. From: The Human Protein Atlas. Bar = 60 µm in (**A**,**D**), and 40 µm in (**B**,**C**).

**Figure 6:**
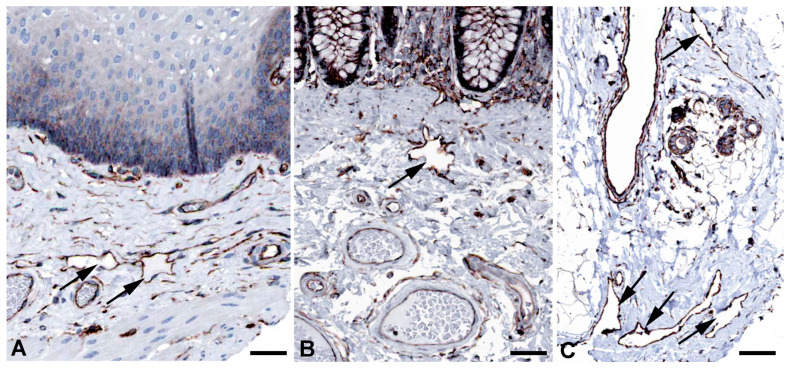
Immunostaining of SPTBN1 in lymphatics (arrows) of human (**A**) Esophagus, Antibody HPA013149, (**B**) Colon, Antibody HPA013149, (**C**) Breast, Antibody HPA013149. From: The Human Protein Atlas. Bar = 50 µm in (**A**,**B**), and 80 µm in (**C**).

Both the initial lymphatic vessels and the lymph collectors exhibit strong functional fluctuations in their diameter, and they have valves whose elasticity is of great importance for reliable valve closure. Spectrins have originally been identified as the major elastic component of erythrocytes, linking the actin cytoskeleton to the cell membrane. However, nonerythroid spectrins have also been identified [33,34,35]. Spectrins form tetrameric proteins of alpha and beta subunits. *SPTAN1* (spectrin, alpha, nonerythrocytic 1) and *SPTBN1* (spectrin, beta, nonerythrocytic 1) are highly expressed in LECs (Table 1) and they are well detectable at protein level in lymphatics of various organs (Figure 5 and Figure 6). Neuropathies related to malfunction of the two molecules have been observed, but lymphedema has not yet been described.

**Figure 7:**
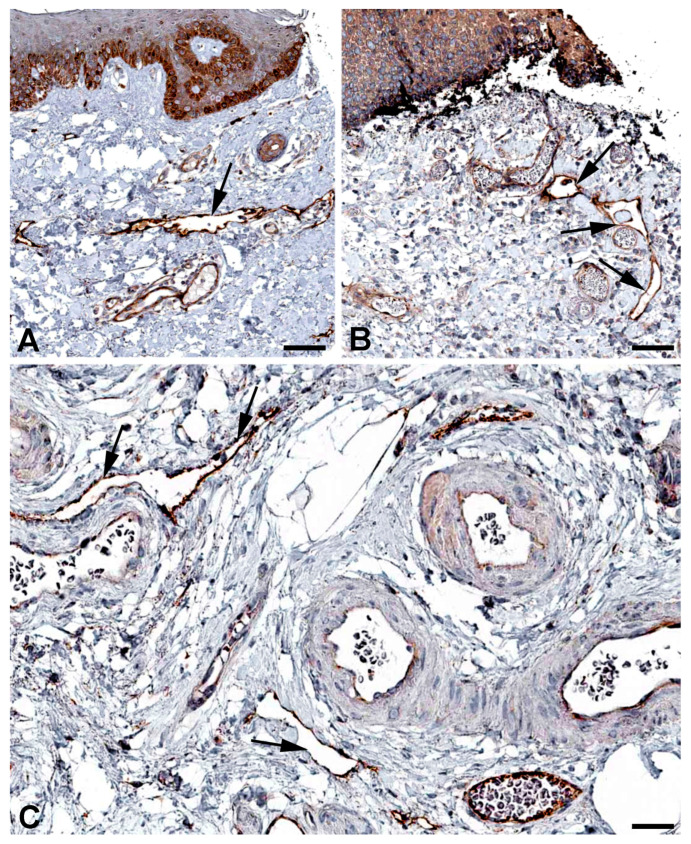
Immunostaining of MMRN1 in lymphatics (arrows) of human (**A**) Skin, Antibody HPA035769, (**B**) Oral mucosa, Antibody HPA035769, (**C**) Urinary bladder, Antibody HPA035769. From: The Human Protein Atlas. Bar = 60 µm in (**A**–**C**).

Among the genes most highly expressed in LECs is Multimerin 1 (*MMRN1*) (Table 1). Lymph contains fibrinogen and can coagulate [36], and we have previously pointed out that LECs are an important source for factor VIII (F8, antihemophilic globulin A) and its carrier protein von-Willebrand factor (VWF) [8]. MMRN1 is a specific coagulation factor V binding platelet protein with a role in hemostasis and coagulation, and accordingly it is highly expressed in megakaryocytes [37]. In addition, it is found with extremely high specificity in endothelial cells; despite this, a clear distinction between BECs and LECs has not been made [38]. However, it appears that LECs are clearly more strongly positive than BECs (https://www.proteinatlas.org/ENSG00000138722-MMRN1/single+cell+type; accessed on 5 November 2024). We found immunopositivity for MMRN1 in lymphatics of various organs (Figure 7). During lymphostasis, the accumulation of pro-coagulatory factors (F8, VWF, MMRN1) and the decrease in the anti-coagulatory and anti-inflammatory 5′-nucleotidase (CD73) of the LECs [39] can be important reasons for the increased tendency to thrombosis and inflammation.

**Figure 8:**
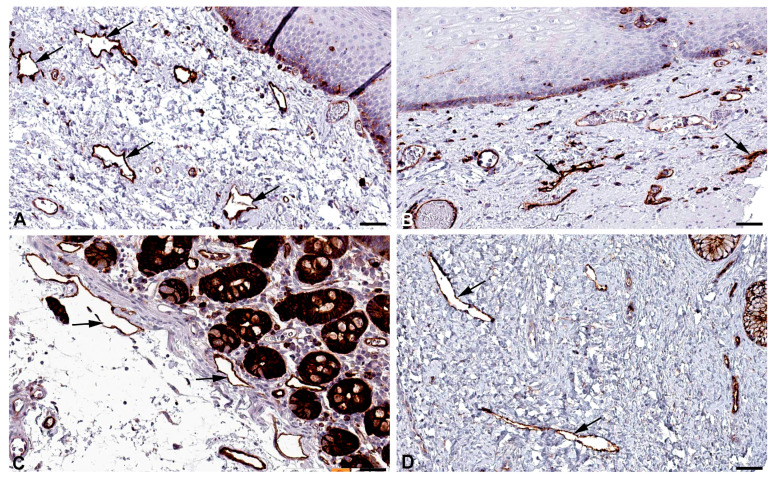
Immunostaining of MARCKSL1 in lymphatics (arrows) of human (**A**) Oral mucosa, Antibody HPA030528, (**B**) Esophagus, Antibody HPA030528, (**C**) Duodenum, Antibody HPA030528, (**D**) Cervix uteri, Antibody HPA030528. From: The Human Protein Atlas. Bar = 55 µm in (**A**,**D**), and 45 µm in (**B**,**C**).

MARCKS-like protein-1 (MARCKSL1 = MLP) has great similarity with the myristoylated, alanine-rich protein MARCKS [40,41], a substrate for protein kinase C. MARCKSL1 has mainly been studied in neural development and in cancer cell migration [42]. High MARCKSL1 expression has a strong prognostic value in lymph node-negative breast cancer patients [43]. Upon phosphorylation, MARCKSL1 induces actin bundling and inhibits cell migration [42]. We observed high RNA expression of *MARCKSL1* in LECs (Table 1) and immune-positive lymphatics in various organs (Figure 8). The influence of MARCKSL1 on LEC stability and function has not yet been studied.

**Figure 9:**
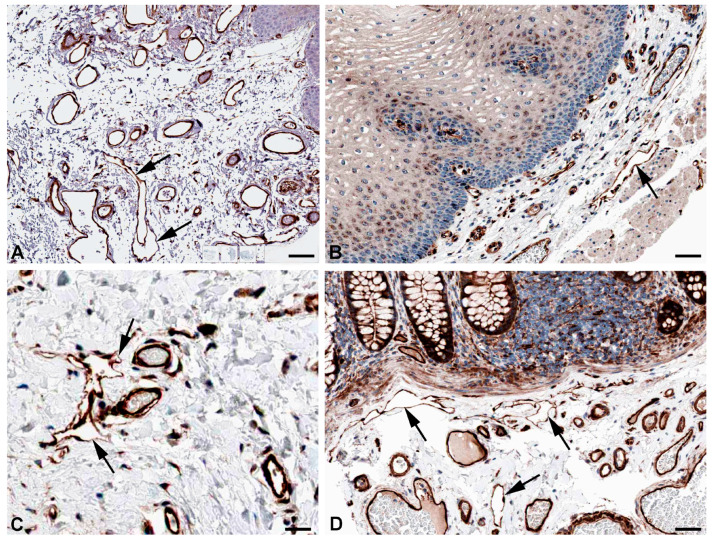
Immunostaining of CNN3 in lymphatics (arrows) of human (**A**) Oral mucosa, Antibody HPA051237, (**B**) Esophagus, Antibody CAB009849, (**C**) Skin, Antibody CAB009849, (**D**) Colon, Antibody CAB009849. From: The Human Protein Atlas. Bar = 70 µm in (**A**), 40 µm in (**B**,**D**), and 30 µm in (**C**).

Another molecule associated with the actin cytoskeletal system is calponin 3 (CNN3). It consists of an acidic C terminus and a basic N terminus [44,45]. CNN3 regulates contractility of actomyosin-containing stress fibers of non-muscle cells [46]. Thereby, control of stress fiber contractility by CNN3 was found to be associated with mechanosensitive Yap/Taz (Yes-associated protein/transcriptional coactivator with PDZ binding motif) transcriptional activation [47]. We observed high RNA expression of *CNN3* in LECs (Table 1) and immune-positive lymphatics in various organs (Figure 9), making LECs an attractive model for further studies on the function of CNN3. The importance of the Yap/Taz signaling in the Hippo pathway for lymphangiogenesis is well recognized [48,49].

**Figure 10:**
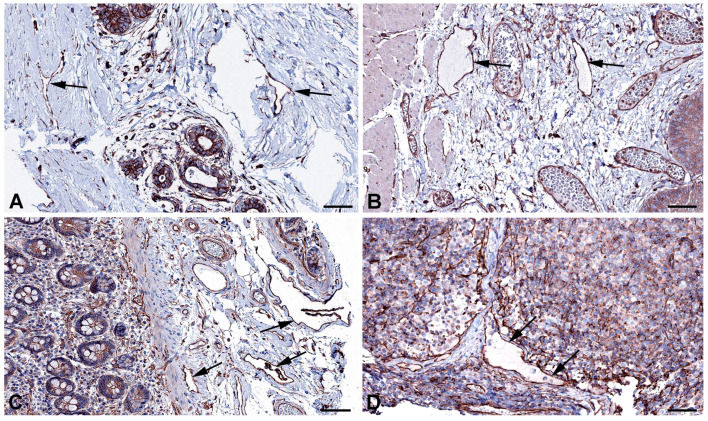
Immunostaining of ANKRD37 in lymphatics (arrows) of human (**A**) Breast, Antibody HPA036626, (**B**) Esophagus, Antibody HPA036626, (**C**) Colon, Antibody HPA036626, (**D**) Lymph node, Antibody HPA036626. From: The Human Protein Atlas. Bar = 80 µm in (**A**–**D**).

The cytoskeleton of LECs appears to be very stable, and accordingly, the invasiveness of LECs is usually extremely low; the only exception being vanishing bone disease (Gorham-Stout disease, GHS) with approx. 350 cases being reported [50,51,52]. The ankyrin repeat domain protein 37 (ANKRD37) is not only associated with preeclampsia during pregnancy. Its knock-down enhances trophoblast invasiveness, migration, and regulation of key invasion proteins [53]. *ANKRD37* is expressed at very low levels in LECs and is significantly upregulated by hypoxia (Table 1) [26]. It is a hypoxia-inducible factor-1 (HIF1) target gene [54]. Its expression in GHS has, to the best of our knowledge, not yet been studied. We observed immune-positivity for ANKRD37 in LECs (Figure 10). However, due to the large number of ANKRD family members expressed in LECs specificity of antibody staining might be problematic.

**Figure 11:**
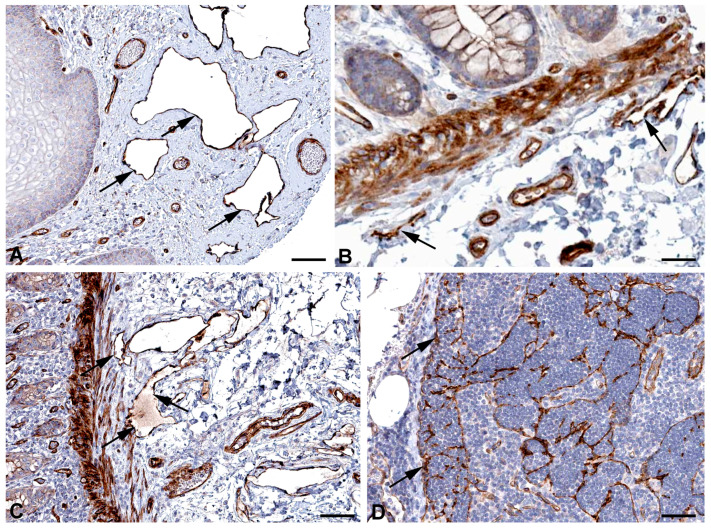
Immunostaining of KANK3 in lymphatics (arrows) of human (**A**) Skin, (obviously edematous), Antibody HPA051153, (**B**) Colon, Antibody HPA051153, (**C**) Rectum, Antibody HPA051153, (**D**) Lymph node, Antibody HPA051153. From: The Human Protein Atlas. Bar = 60 µm in (**A**,**C**), 40 µm in (**B**), and 80 µm in (**D**).

Another molecule involved in actin stress fiber formation and containing an ankyrin repeat domain is KANK3 (KN motif- and ankyrin repeat domain-containing protein 3) [55]. *KANK3* is highly expressed in LECs (Table 1) and immune-positivity is seen in lymphatics of various organs (Figure 11). The four members of the KANK family regulate integrin-mediated adhesion, actomyosin contractility, and link focal adhesions to the cortical microtubule stabilization complex [56]. Specific expression of KANK3 in endothelial cells has been noted [56], but functional studies are lacking.

**Figure 12:**
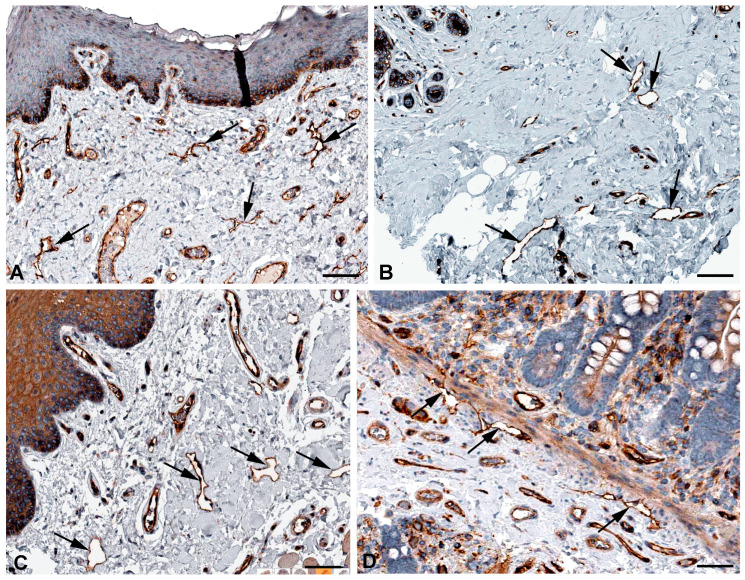
Immunostaining of CD59 in lymphatics (arrows) of human (**A**) Skin, Antibody HPA026494, (**B**) Breast, Antibody HPA0264949, (**C**) Oral mucosa, Antibody HPA026494, (**D**) Colon, Antibody HPA026494. From: The Human Protein Atlas. Bar = 80 µm in (**A**,**C**), 100 µm in (**B**), and 50 µm in (**D**).

Lymph contains high amounts of immunoglobulin G (IgG) heavy chain [36]. Antigen-IgG complexes represent a starting point for the classical complement system, which can stimulate phagocytes, inflammation, and the cell-killing membrane attack complex (MAC) [57]. LECs express high amounts of *CD59*, also known as Protectin (Table 1), a glycoprotein functioning as a membrane-bound inhibitor of MAC [58]. Malfunctioning of CD59 causes hemolytic anemia with immune-mediated polyneuropathy [59]. The specific function in LECs has not been investigated yet. Immune-positivity is seen in lymphatics and blood vessels (Figure 12), suggesting an important immune-suppressive function in vessels. Strong expression of CD59 in colorectal cancer is associated with higher incidence of lymph node metastasis [55]. In ulcerative colitis, a chronic inflammatory disease of the colon, CD59 belongs to the complement components that have been shown to safeguard the intestinal barrier and reduce intestinal inflammation [60].

**Figure 13:**
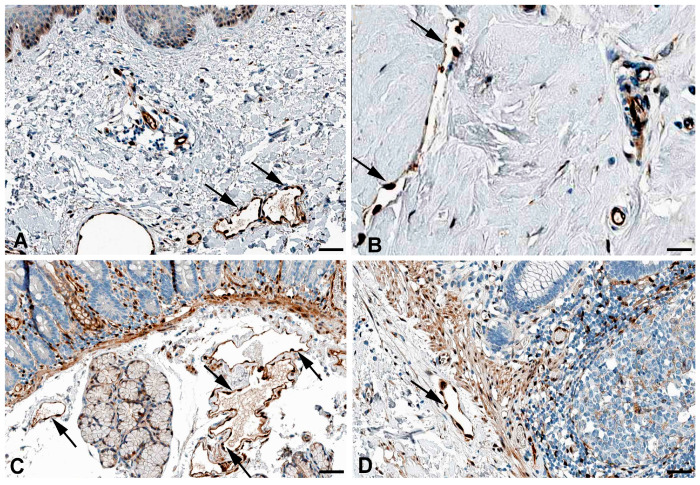
Immunostaining of NXN in lymphatics (arrows) of human (**A**) Skin, Antibody HPA023566, (**B**) Breast, Antibody HPA023566, (**C**) Duodenum, Antibody HPA023566, (**D**) Colon, Antibody HPA023566. From: The Human Protein Atlas. Bar = 60 µm in (**A**,**C**), 25 µm in (**B**), and 40 µm in (**D**).

It was previously shown that the non-canonical WNT (Wingless-type MMTV integration site) signaling pathway is of great importance for the elongation of lymphatics during embryonic lymphangiogenesis [61,62]. Nucleoredoxin (NXN) is a 48 kDa protein and a redox-dependent negative regulator of the Wnt signaling pathway [63]. *NXN* mRNA is highly expressed in LECs (Table 1), and it can be detected in lymphatics of various organs by immunohistology (Figure 13). Similar to mutations in *WNT5A* [64] and other WNT signaling members, mutations in *NXN* cause Robinow syndrome [65], which is mainly characterized by dysmorphic facial features and short-limbed dwarfism. To our knowledge, morphology and function of the lymphatics have not yet been investigated in Robinow patients.

## 3. Materials and Methods

### 3.1. Cell Culture

We used three well characterized human dermal lymphatic endothelial cell lines (PromoCell, Heidelberg, Germany). Cells and culturing were described before [10,26].

### 3.2. RNA Sequencing

RNASeq of defined lymphatic endothelial cells was performed as described [10,26].

### 3.3. Immunofluorescence (IF)

IF was performed on human foreskin derived from operations performed at the University Medical Center Goettingen (UMG). Specimens were fixed in 4% paraformaldehyde for 1 h, embedded in tissue freeze medium, and sectioned at 12 µm. Studies were performed with the informed consent of the patients or their parents and were approved by the ethics committee of the UMG (application no. 18/1/18). Primary antibodies were mouse-anti-human CD31 (BD Pharmingen, Franklin Lakes, NJ, USA, dilution 1:50, Lot: 550389) and rabbit-anti-human dysferlin (Sigma, St. Louis, MO, USA, dilution 1:200, Lot: 19895). Secondary antibodies were Alexa 488-conjugated goat-anti-mouse IgG (H + L) (Invitrogen, Waltham, MA, USA, dilution 1:200, Lot: 2765658) and Alexa 594-conjugated goat-anti-rabbit IgG (H + L) (Invitrogen, dilution 1:200, Lot: 2506100). Nuclei were counterstained with Dapi (Invitrogen).

### 3.4. Immunohistochemistry

We compared our RNASeq expression data with protein expression by systematically studying the Human Protein Atlas [27] (https://www.proteinatlas.org/; accessed on 5 November 2024). All figures shown here can be found and further studied at variable magnification in this repository.

## 4. Conclusions

The exome of lymphatic endothelial cells is very extensive, as in other cell types. The correspondence with the protein expression that we have studied in the Human Protein Atlas is comparatively low. However, this can probably be attributed to simple technical reasons when studying paraffin-embedded human tissues. The selected images show preparations of both the body wall (skin, breast) and internal organs. This indicates that the selected molecules may be important in LECs of somatic and visceral origin. We found good agreement for molecules associated with the high transcytosis activity of LECs (caveolin, dysferlin). There was very good agreement for molecules that have important functions in the structure and regulation of the cytoskeleton. This may be due to the fact that the LECs of the initial lymphatic vessels have a unique morphology and function. This is expressed in the oak leaf-like morphology of the cells with the formation of specialized button-like junctions and microvalves, which are of essential importance for the directed lymph flow [66,67,68]. Accordingly, we found expression of molecules that mediate cellular elasticity (spectrins) and interact with actin microfilaments (ANKRD37, CNN3, KANK3, MARCKSL1). In terms of immune regulation, lymphatic vessels are a double-edged sword [8]. They are the main route for the emigration of leukocytes towards the lymph nodes. However, they can also have an immunosuppressive effect, which is reflected in the expression of CD59. The influence of LECs on coagulation, which has already been described several times, was confirmed in our investigations (multimerin1). The importance of the WNT signaling pathway for development and function of lymphatics still needs to be investigated in greater detail (NXN). In sum, we hope our studies can stimulate further studies on the complex morphology and functions of lymphatics.

## Figures and Tables

**Table 1:** RNASeq analysis of three defined human dermal LEC lines under normoxia and hypoxia.

Gene_ID	Gene-Name	Chromos.	Start	End	Width	Strand	Gene_Bio-Type	HDLEC-5	HDLEC-6	HDLEC-7	Hypox-5	Hypox-6	Hypox-7
ENSG00000186352	*ANKRD37*	4	1.85 × 10^8^	1.85 × 10^8^	4608	+	protein_cod.	93	117	124	312	549	650
ENSG00000105974	*CAV1*	7	1.17 × 10^8^	1.17 × 10^8^	36,184	+	protein_cod.	28,254	22,816	40,041	41,886	29,070	44,229
ENSG00000105971	*CAV2*	7	1.16 × 10^8^	1.17 × 10^8^	221,162	+	protein_cod.	5923	6362	8339	8258	7425	10,628
ENSG00000085063	*CD59*	11	33,703,010	33,736,491	33,482	−	protein_cod.	25,997	30,542	33,420	25,878	36,425	34,222
ENSG00000117519	*CNN3*	4	94,896,949	94,927,223	30,275	−	protein_cod.	16,690	24,868	11,454	14,357	20,711	8938
ENSG00000135636	*DYSF*	2	71,453,722	71,686,768	233,047	+	protein_cod.	2656	1698	4385	3354	2205	5193
ENSG00000186994	*KANK3*	19	8,322,584	8,343,262	20,679	−	protein_cod.	2257	4008	2949	3946	4202	5177
ENSG00000175130	*MARCKSL1*	1	32,333,839	32,336,233	2395	−	protein_cod.	8786	9698	8004	8408	7819	4811
ENSG00000138722	*MMRN1*	4	89,879,532	89,954,629	75,098	+	protein_cod.	199,494	204,322	16,869	263,839	303,443	38,908
ENSG00000138119	*MYOF*	10	93,306,429	93,482,334	175,906	−	protein_cod.	12,506	4987	5959	11,865	2877	11,681
ENSG00000167693	*NXN*	17	799,310	979,776	180,467	−	protein_cod.	5647	7278	2263	4813	5743	2401
ENSG00000115155	*OTOF*	2	26,457,203	26,558,698	101,496	−	protein_cod.	1187	1088	464	499	109	146
ENSG00000197694	*SPTAN1*	9	1.29 × 10^8^	1.29 × 10^8^	81,105	+	protein_cod.	10,798	8181	6610	8411	9524	8409
ENSG00000115306	*SPTBN1*	2	54,456,317	54,671,446	215,130	+	protein_cod.	23,119	28,282	11,418	25,384	39,160	14,477

Three human dermal lymphatic endothelial cell lines (HDLEC-5, 6, and 7) were investigated under 21% pO_2_ as well as under 1% pO_2_ (Hypox-5, 6, and 7). RNASeq was performed by the NGS-Integrative Genomics Core Unit, UMG, Göttingen (details see: [10]. The number of reads is presented. The molecules shown here are not regulated by hypoxia with two exceptions: Otoferlin (OTOF), which is a moderately high expressed gene, is downregulated by hypoxia. OTOF belongs to the ferlin family of proteins, additionally including dysferlin (DYSF) and myoferlin (MYOF). ANKRD37 is a lowly expressed gene, which is upregulated by hypoxia.

## Data Availability

All data are included in the manuscript. Additional immunohistochemical data are found at: The Human Protein Atlas (https://www.proteinatlas.org/; accessed on 5 November 2024).

## Abbreviations

ANKRD37—ankyrin repeat domain protein 37; BEC—Blood vascular endothelial cell; CAV1/2—Caveolin 1/2; CD59—CD59 glycoprotein aka Protectin; CNN3—Calponin 3; DYSF—Dysferlin; GHS—Gorham-Stout disease/syndrome; KANK3—KN motif- and ankyrin repeat domain-containing protein 3; LEC—Lymphatic endothelial cell; MAC—Membrane attack complex; MARCKSL1—MARCKS-like protein-1; MMRN1—Multimerin 1; MYOF—Myoferlin; NXN—Nucleoredoxin; OTOF—Otoferlin; SPTAN1—Spectrin, alpha, nonerythrocytic 1; SPTBN1—Spectrin, beta, nonerythrocytic 1; WNT—Wingless-type MMTV integration site.
